# Clinical evaluation of the biological width following surgical crown-lengthening procedure: A prospective study

**DOI:** 10.4103/0972-124X.75910

**Published:** 2010

**Authors:** K. S. Shobha, Mahantesha, Hema Seshan, R. Mani, K Kranti

**Affiliations:** *Sri Siddhartha Dental College and Hospital, Tumkur, India*; 1 *Department of Periodontics, K.L.E Society’s Institute of Dental Sciences, Bangalore, Karnataka, India*; 2 *Department of Periodontics, M.S. Ramaiah Dental College and Hospital, Bangalore, Karnataka, India*

**Keywords:** Biological width, crown-lengthening, osseous resection

## Abstract

**Background and Objectives:**

The purpose of this study was to evaluate the positional changes of the periodontal tissues, particularly the biological width, following surgical crown-lengthening in human subjects.

**Materials and Methods:**

A clinical trial study involving 15 patients was carried out for a period of 6 months. Sites were divided into 3 groups: treated (TT) sites, adjacent (AD) sites and nonadjacent (NAD) sites. Free gingival margin [FGM], attachment level, pocket depth, bone level, biological width [BW] were recorded at baseline, 1, 3 and 6 months. Direct bone level after flap reflection was recorded before and after osseous resection at baseline only. Level of osseous crest was lowered based on BW, and supracrestal tooth structure needed using a combination of rotary and hand instruments.

**Statistical Analysis:**

Student *t* test and ANOVA were used.

**Results:**

Overall, apical displacement of FGM at TT, AD and NAD sites was statistically significant compared to baseline. The apical displacement of FGM at TT site was more when compared to that at AD and NAD sites at 3 and 6 months. The BW at the TT site was smaller at 1, 3 and 6 months compared to that at baseline. However, at all sites, BW was reestablished to the baseline value at the end of 6 months.

**Interpretation and Conclusion:**

The BW at TT sites was reestablished to its original vertical dimension by 6 months. In addition, a consistent 2-mm gain of coronal tooth structure was observed at the 1, 3 and 6-month examinations.

## INTRODUCTION

Periodontal tissues form the foundation for proper esthetics, function and comfort of the dentition.[1] Biological width acts as a barrier to prevent penetration of microorganisms into the periodontium.[2]

Restoration of a tooth without regard to the biological width results in poor periodontal response.[3] Surgical crown-lengthening provides enough sound tooth structure by reestablishing a healthy periodontium, particularly the biological width at a more apical level.[4]

The present study was undertaken to clinically evaluate the positional changes of periodontal tissues and biological width following surgical crown-lengthening procedure at the treated, adjacent and nonadjacent sites at baseline, 1, 3 and 6 months.

## MATERIALS AND METHODS

For this study, 15 to 20 patients requiring surgical crown-lengthening were selected from the Outpatient Department of Periodontics, M. S. Ramaiah Dental College, Bangalore, Karnataka. It was a clinical trial study.

### Inclusion criteria

- Patients in the age group of 15 to 72 years
- Patients with adequate width of attached gingiva
- Patients requiring surgical crown-lengthening due to any of the following:
  - Delayed passive eruption
  - Requiring subgingival restorations
  - Lack of retention for crown placement
  - Subgingival caries
  - Subgingival crown margins or root fracture, root perforations
  - Gummy smile
  - Gingival margin discrepancies
  - Short clinical crowns with high lip (smile) line

### Exclusion criteria

- Grade II/ III mobile teeth
- Periodontal pockets of ≥4 mm
- Bone loss
- Unrestorable teeth
- Local or systemic contraindications to surgery
- Molars with less-than-adequate periodontal support and furcation involvement

### Study design

Fifteen patients were selected after the initial phases of periodontal therapy. Patients were given a brief description of the study, and a written informed consent letter was obtained from all the patients. The selected sites were divided into 3 groups:

Treated (TT) sites: Sites on teeth selected for crown-lengthening

Adjacent (AD) sites: Interproximal sites that shared a proximal surface with the treated tooth

Nonadjacent (NAD) sites: Interproximal sites away from the treated tooth

#### Clinical parameters

**Figure 1:**
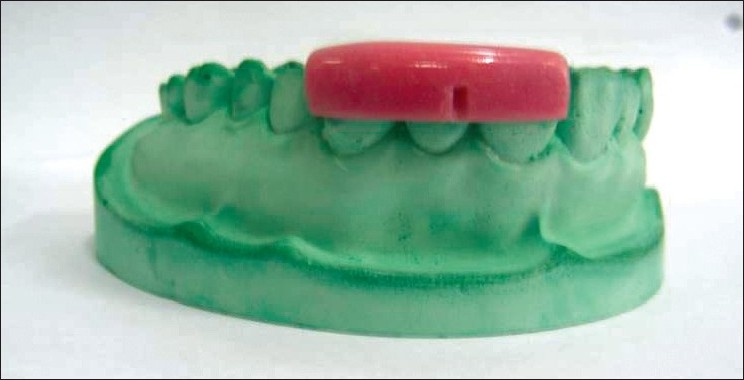
Stent with groove for vertical probing

**Figure 2:**
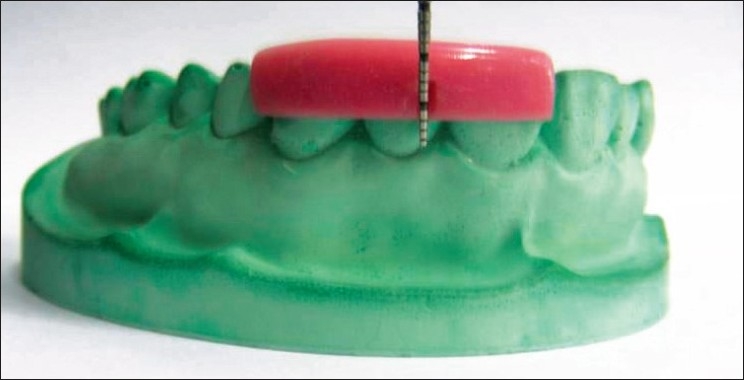
Stent with PCP-UNC–15 probe in place

All the measurements were standardized using customized acrylic stents with grooves and recorded using a University of North Carolina probe (PCP-UNC–15 probe. (Hu-Friedy’s) [Figures 1 and 2].

**Figure 3:**
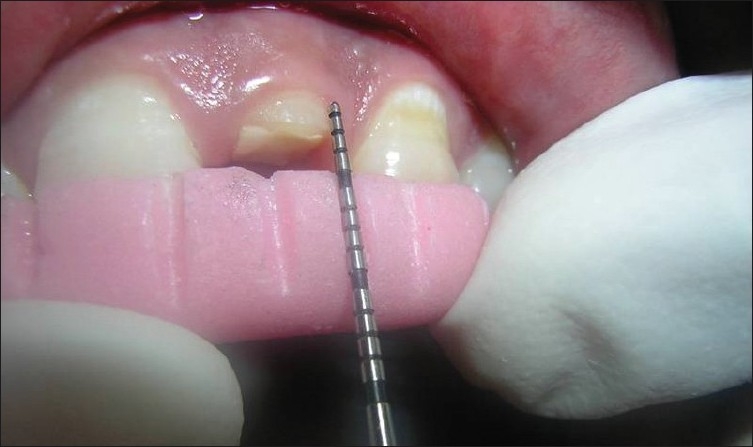
Free gingival margin measurement at the treated site (baseline)

**Figure 4:**
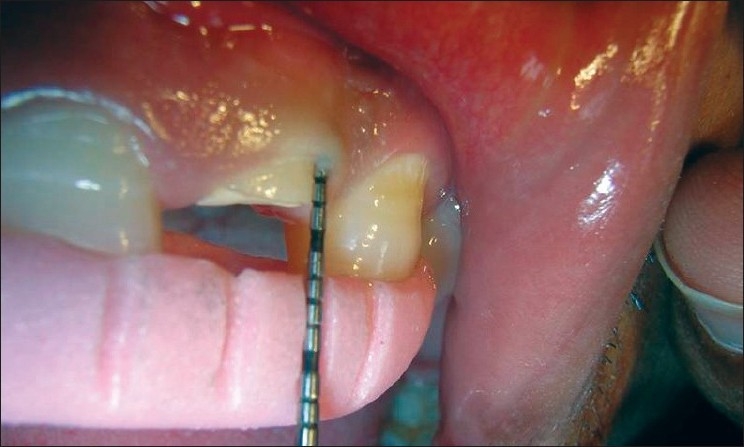
Attachment level measurement at the treated site (baseline)

**Figure 5:**
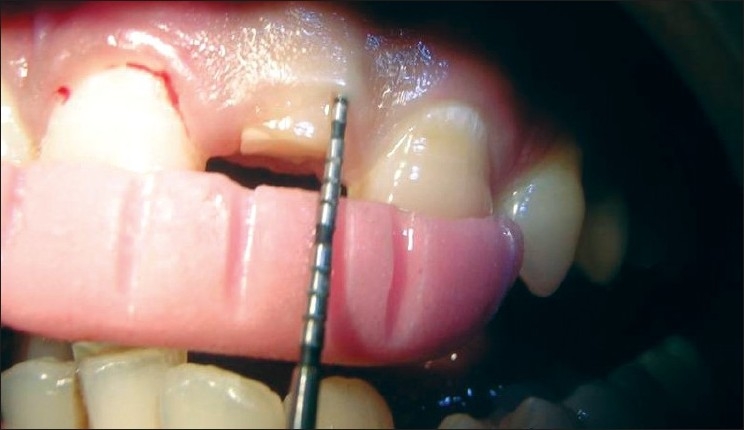
Bone level measurement at the treated site (baseline)

**Figure 6:**
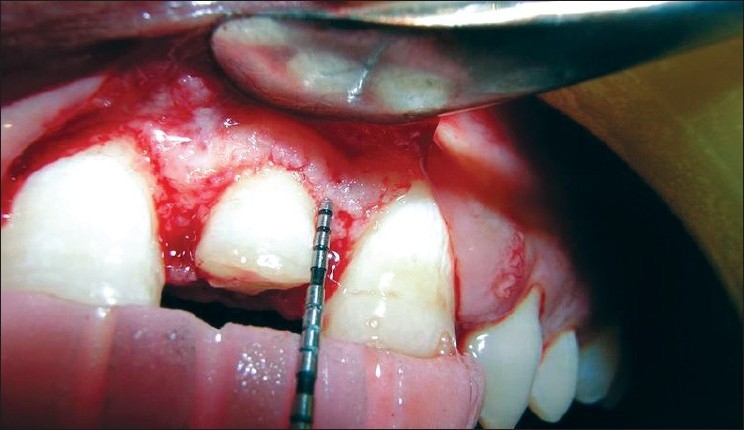
Direct bone level measurement before osseous resection at the treated site

**Figure 7:**
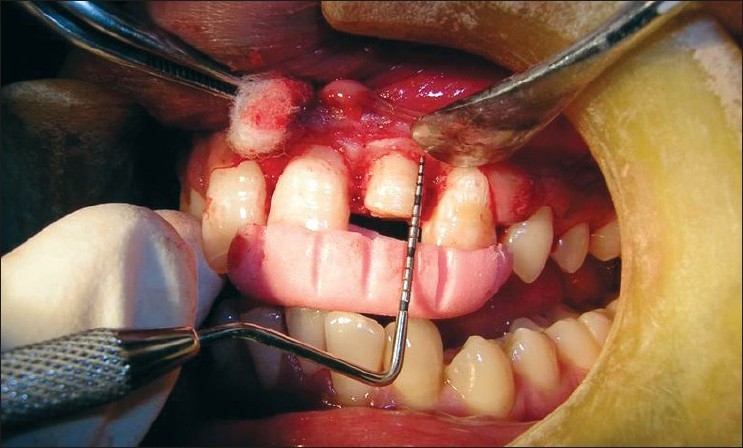
Direct bone level measurement after osseous resection at the treated site

The following clinical parameters were recorded at 4 sites — mesiobuccal, mesiolingual, distobuccal and distolingual — around every TT, AD and NAD site at baseline [Figures 3–7], 1, 3 and 6 months:

- Plaque index (Silness and Loe, 1964)
- Gingival index (Loe and Silness, 1963)
- Distance from the fixed reference point (FRP) to the free gingival margin (FGM)
- Distance from the fixed reference point (FRP) to the attachment level (AL) of the base of the pocket (BOP)
- After the experimental area was anesthetized, bone level (BL) was obtained via transgingival probing from the fixed reference point
- The direct bone level, viz., the distance from the fixed reference point to the bone level, was measured after reflection of the flap, before and after ostectomy

The lower/apical limit of the vertical grooves was used as the fixed reference point for the vertical probing depths (Samuel E. Lynch, 1992). The following calculations were made from the clinical measurements recorded:

- Probing depth
  (FRP to BOP) – (FRP to FGM)
- Biological width
  (FRP to BL) – (FRP to BOP)

#### Pre-surgical phase

Selected patients were subjected to phase I periodontal therapy. For each patient, the magnitude of biological width was added to the amount of supracrestal tooth structure needed to be exposed for various treatments. The level of osseous crest was lowered based on this amount.

#### Armamentarium

- UNC-15 graduated periodontal probe (Hu-Friedy’s)
- Straight probe
- Disposable syringe
- Local anesthetic (2% Xylocaine HCL with adrenaline 1:80,000)
- Bard-Parker (B.P) handles with no. 11, 12 and 15 blades
- Periosteal elevator
- Gracey curettes and universal curettes
- Scissors
- Needle holder
- 3/8 reverse-cutting swaged needle and 3-0 black braided silk suture
- Cotton swabs
- Kidney tray with saline and irrigation syringe
- Straight fissure– and round bone–cutting burs
- Straight micro-Z hand piece and cord

#### Surgical procedure

After anesthesia, a modified Widman incision was made with a bard parker no. 11 on the tooth requiring crown-lengthening. After excision of the incised tissue, a sulcular incision was made on the adjacent teeth mesially and distally with a bard parker no. 15.

A full-thickness mucoperiosteal flap was raised. Degranulation and thorough root planning was done. The level of osseous crest was lowered based on the calculation using a combination of straight fissure and round bur under saline irrigation. Direct bone levels were measured before and after ostectomy.

**Figure 8:**
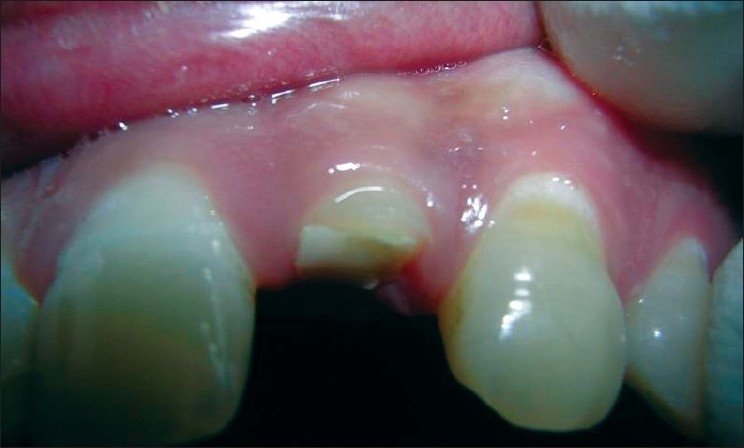
Preoperative view

**Figure 9:**
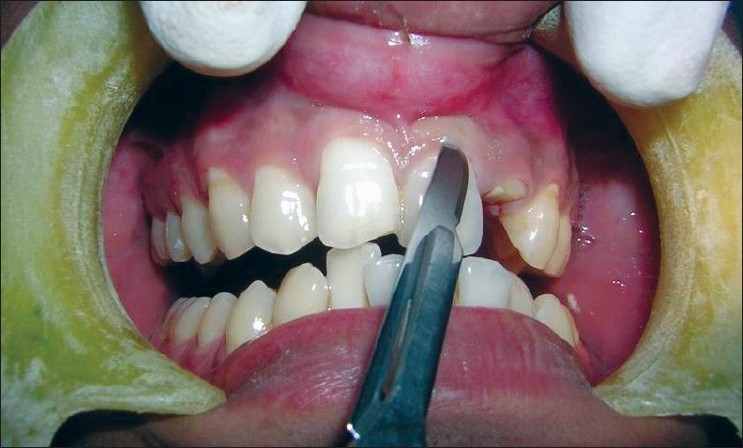
Incision

**Figure 10:**
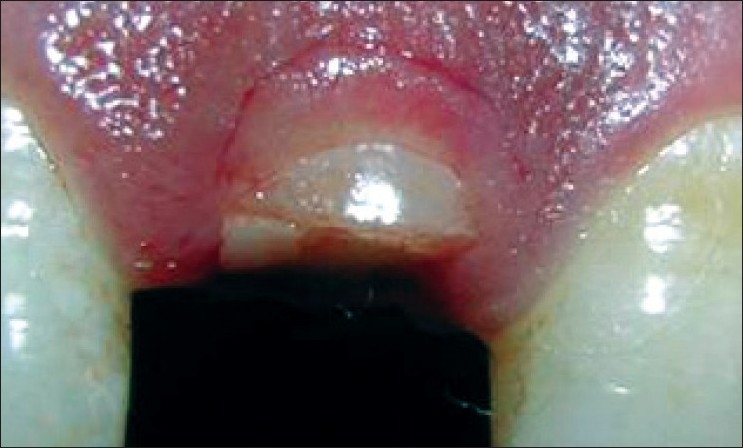
Incision made

**Figure 11:**
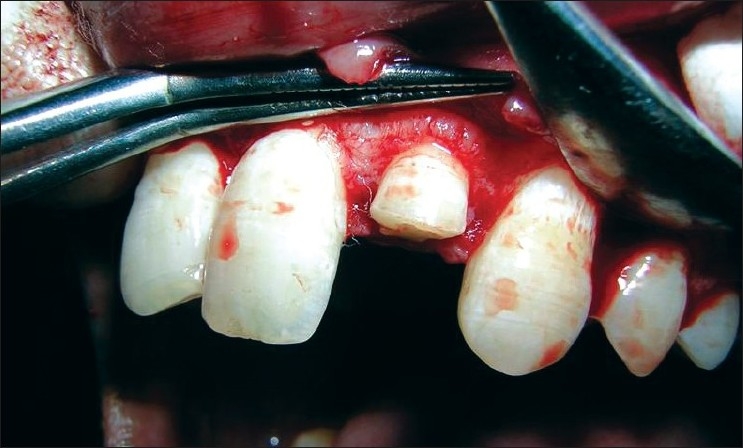
Full-thickness flap elevated

**Figure 12:**
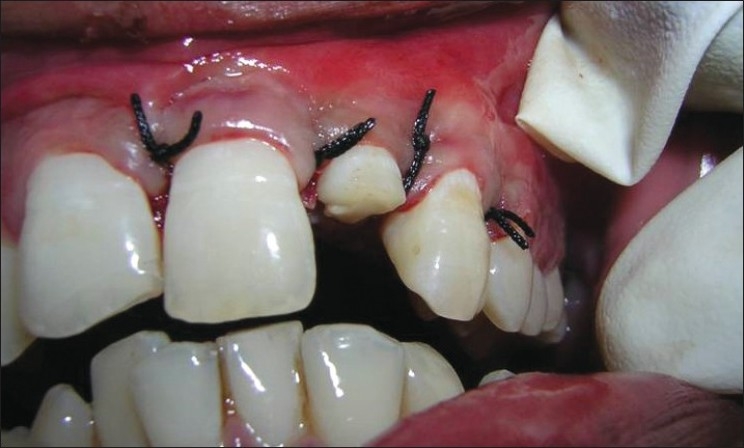
Sutures placed

**Figure 13:**
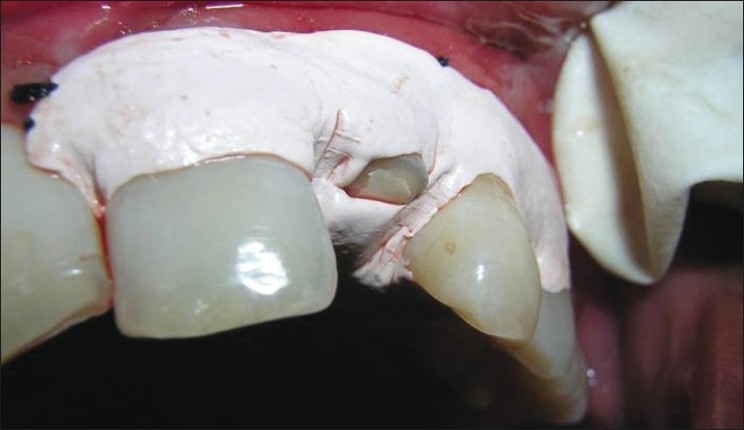
Periodontal dressing

Flaps were sutured using 3-0 black braided silk, and periodontal dressing was placed [Figures 8–13]. Amoxicillin 500 mg, thrice daily for 5 days; ibuprofen 400 mg and Paracetamol 325 mg, twice daily for the next 5 days were prescribed. Patients were given postoperative instructions and were instructed to report after 24 hours of surgery and after 7 days.

#### Postoperative instructions

**Figure 14:**
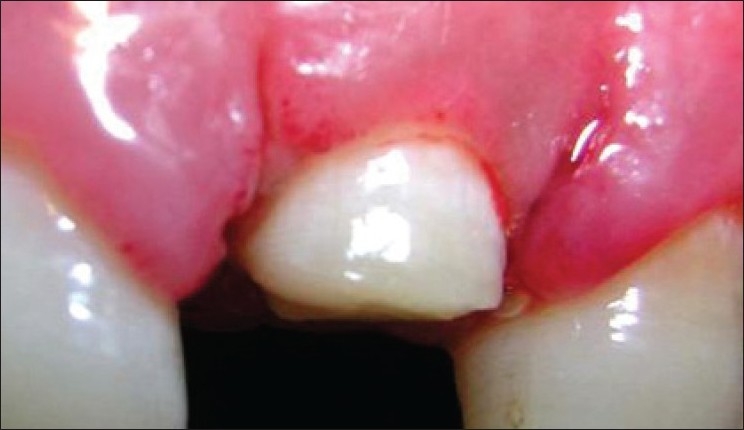
One-week postoperative

At 7 days following surgeries, the dressing and sutures were removed [Figure 14]. After 1 month, the stability of the gingival margin was evaluated. If found stable and free of inflammation, then they were referred for crown restoration.

#### Post-surgical evaluation

##### Clinical evaluation

**Figure 15:**
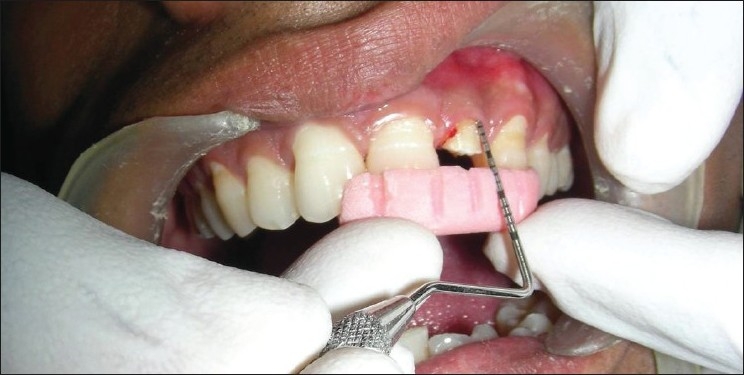
Free gingival margin measurement at the treated site (at 6 months)

**Figure 16:**
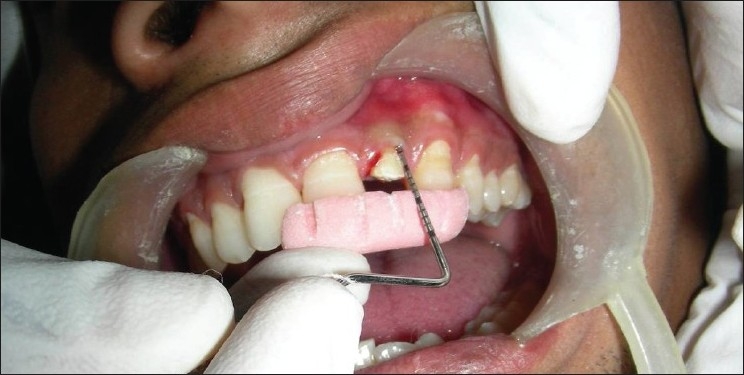
Attachment level measurement at the treated site (at 6 months)

**Figure 17:**
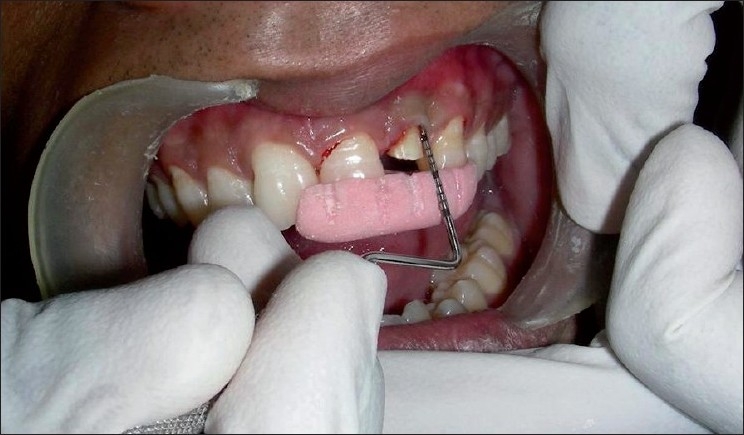
Bone level measurement at the treated site (at 6 months)

**Figure 18:**
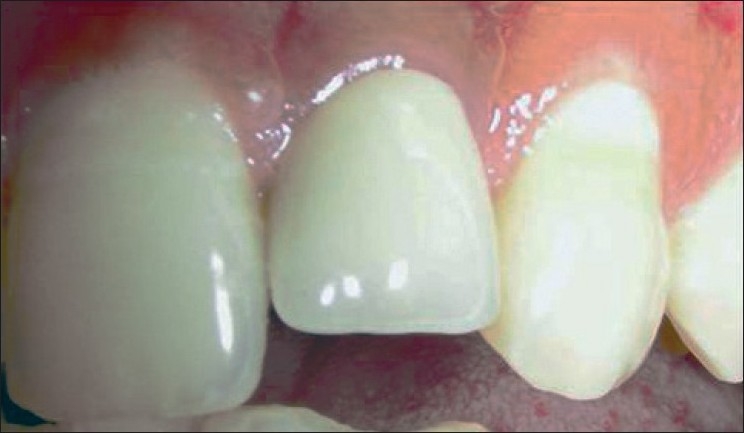
Ceramic crown

The patients were evaluated clinically at 1, 3 and 6 months post-surgery [Figures 15–18].

### Method of statistical analysis

The following methods of statistical analysis were used in this study.

The results for each average (mean±standard deviation) for continuous data are presented in Tables 1–13.

**Table 1:** Mean values of FGM at baseline, 1 month, 3 months and 6 months

	Treated site		Adjacent site		Nonadjacent site	
	Mean	Std. dev.	Mean	Std. dev.	Mean	Std. dev.
Baseline	1.93	1.03	3.27	1.28	2.93	1.16
1 Month	4.20	1.74	4.33	1.40	4.07	1.44
3 Months	4.27	1.53	4.20	1.15	3.93	1.39
6 Months	4.20	1.66	4.13	1.19	3.93	1.33

**Table 2:** Comparison of change in FGM from baseline to 6 months

	Treated site				Adjacent site				Nonadjacent site			
	Mean	Std. dev.	*t* value	Pr>*t*	Mean	Std. dev.	*t* value	Pr>*t*	Mean	Std. dev.	*t* value	Pr>*t*
1 Month	2.27	0.80	10.99	<.0001(HS)	1.07	0.59	6.96	<.0001(HS)	1.13	0.83	5.26	0.0001(HS)
3 Months	2.33	0.72	12.49	<.0001(HS)	0.93	0.70	5.14	0.0002(S)	1.00	0.93	4.18	0.0009(S)
6 Months	2.27	0.80	10.99	<.0001(HS)	0.87	0.83	4.03	0.0013(S)	1.00	1.07	3.62	0.0028(S)

*P*≥.05 (not significant, NS); *P*≤.05 (significant, S); *P*≤.0001 (highly significant, HS)

**Table 3:** Mean values of AL at baseline, 1 month, 3 months and 6 months

	Treated site		Adjacent site		Nonadjacent site	
	Mean	Std. dev.	Mean	Std. dev.	Mean	Std. dev.
Baseline	4.80	1.26	5.07	1.03	5.00	1.25
1 Month	6.20	1.42	6.20	1.37	5.73	1.49
3 Months	6.40	1.30	5.87	1.13	5.93	1.16
6 Months	6.13	1.30	5.87	0.92	5.87	1.30

**Table 4:** Comparison of change in AL from baseline to 6 months

	Treated site				Adjacent site				Nonadjacent site			
	Mean	Std. dev.	*t* value	Pr>*t*	Mean	Std. dev.	*t* value	Pr>*t*	Mean	Std. dev.	*t* value	Pr>*t*
1 Month	1.40	0.91	5.96	<.0001(HS)	1.13	0.74	5.91	<.0001(HS)	0.73	0.70	4.04	0.0012(S)
3 Months	1.60	0.99	6.29	<.0001(HS)	0.80	0.77	4.00	0.0013(S)	0.93	0.88	4.09	0.0011(S)
6 Months	1.33	0.82	6.32	<.0001(HS)	0.80	0.77	4.00	0.0013(S)	0.87	0.92	3.67	0.0025(S)

*P*≥.05 (Not significant = NS); *P*≤.05 (Significant = S); *P*≤.0001 (Highly significant = HS)

**Table 5:** Mean values of probing depth (PD) at baseline, 1 month, 3 months and 6 months

FD	Treated site		Adjacent site		Nonadjacent site	
	Mean	Std. dev.	Mean	Std. dev.	Mean	Std. dev.
Baseline	2.87	1.06	1.87	0.74	1.93	0.80
1 Month	1.87	0.92	1.93	0.70	1.73	0.80
3 Months	1.93	0.80	1.67	0.82	2.00	0.85
6 Months	1.93	0.88	1.73	0.80	1.93	0.70

**Table 6:** Comparison of change in probing depth (PD) from baseline to 6 months

	Treated site				Adjacent site				Nonadjacent site			
	Mean	Std. dev.	*t* value	Pr>*t*	Mean	Std. dev.	*t* value	Pr>*t*	Mean	Std. dev.	*t* value	Pr>*t*
1 Month	–1.00	0.76	–5.12	0.0002(S)	0.07	0.70	0.37	0.7192(NS)	–0.20	0.86	–0.90	0.3840(NS)
3 Months	–0.93	0.88	–4.09	0.0011(S)	–0.20	0.68	–1.15	0.2711(NS)	0.07	1.28	0.20	0.8430(NS)
6 Months	–0.93	0.88	–4.09	0.0011(S)	–0.13	0.74	–0.69	0.4985(NS)	0.00	1.07	0.00	1.0000(NS)

*P*≥.05 (not significant, NS); *P*≤.05 (significant, S); *P*≤.0001 (highly significant, HS)

**Table 7:** Mean values of bone level (BL) at baseline, 1 month, 3 months and 6 months

	Treated site		Adjacent site		Nonadjacent site	
	Mean	Std. dev.	Mean	Std. dev.	Mean	Std. dev.
Baseline	6.60	1.30	6.60	0.91	6.67	1.05
1 Month	7.93	1.33	8.13	0.83	7.67	1.05
3 Months	8.07	0.80	7.87	0.92	7.80	0.86
6 Months	8.00	0.93	7.47	0.83	7.60	1.12

**Table 8:** Comparison of change in bone level (BL) from baseline to 6 months

	Treated site				Adjacent site				Nonadjacent site			
	Mean	Std. dev.	*t* value	Pr>*t*	Mean	Std. dev.	*t* value	Pr>*t*	Mean	Std. dev.	*t* value	Pr>*t*
1 Month	1.33	0.90	5.74	<.0001(HS)	1.53	0.64	9.28	<.0001(HS)	1.00	0.76	5.12	0.0002(S)
3 Months	1.47	0.92	6.20	<.0001(HS)	1.27	0.70	6.97	<.0001(HS)	1.13	0.92	4.79	0.0003(S)
6 Months	1.40	0.99	5.50	<.0001(HS)	0.87	0.64	5.25	0.0001(HS)	0.93	0.96	3.76	0.0021(S)

*P*≥.05 (not significant, NS); *P*≤.05 (significant, S); *P*≤.0001 (highly significant, HS)

**Table 9:** Mean values of BW at baseline, 1 month, 3 months and 6 months

	Treated site		Adjacent site		Nonadjacent site	
	Mean	Std. dev.	Mean	Std. dev.	Mean	Std. dev.
Baseline	1.80	0.77	1.53	0.74	1.73	0.88
1 Month	1.73	0.59	1.93	1.03	1.93	0.88
3 Months	1.67	0.62	1.93	0.80	1.87	0.64
6 Months	1.87	0.83	1.60	0.51	1.80	0.56

**Table 10:** Comparison of change in BW from baseline to 6 months

	Treated site				Adjacent site				Nonadjacent site			
	Mean	Std.dev.	*t* value	Pr>*t*	Mean	Std. dev.	*t* value	Pr>*t*	Mean	Std. dev.	*t* value	Pr>*t*
1 Month	–0.07	0.88	–0.29	0.7744(NS)	0.40	1.06	1.47	0.1643(NS)	0.20	0.86	0.90	0.3840(NS)
3 Months	–0.13	1.06	–0.49	0.6337(NS)	0.40	0.74	2.10	0.0541(NS)	0.13	0.99	0.52	0.6102(NS)
6 Months	0.07	1.10	0.23	0.8178(NS)	0.07	0.80	0.32	0.7513(NS)	0.07	1.03	0.25	0.8062(NS)

*P*≥.05 (not significant, NS); *P*≤.05 (significant, S); *P*≤.0001 (highly significant, HS)

**Table 11:** Bone level (BL) and direct bone level (DBL) measurements at treated, adjacent and nonadjacent sites

	Treated site		Adjacent site		Nonadjacent site	
	Mean	Std. dev.	Mean	Std. dev.	Mean	Std. dev.
BL at baseline	6.40	1.24	6.53	0.92	6.67	1.05
DBL Immd. before osseous reduction	6.33	1.35	6.47	0.99	6.33	0.90
DBL Immd. after osseous reduction	7.93	1.16	7.53	1.06	7.33	0.90
Bone level at 3 months	8.07	0.80	7.87	0.92	7.80	0.86

**Table 12:** Comparison of change in direct bone level (DBL) from baseline to 3 months

	Treated site				Adjacent Site				Nonadjacent site			
	Mean	Std. dev.	*t* value	Pr>*t*	Mean	Std. dev.	*t* value	Pr>*t*	Mean	Std. dev.	*t* value	Pr>*t*
Immd. before osseous resection	–0.07	0.80	–0.32	0.7513(NS)	–0.07	0.59	–0.43	0.6702(NS)	–0.33	0.72	–1.78	0.0961(NS)
Immd. after osseous resection	1.53	0.74	7.99	<.0001(HS)	1.00	0.65	5.92	<.0001(HS)	0.67	0.72	3.57	0.0031(S)
3 Months	1.67	0.90	7.17	<.0001(HS)	1.33	0.62	8.37	<.0001(HS)	1.13	0.92	4.79	0.0003(S)

*P*≥.05 (not significant, NS); *P*≤.05 (significant, S); *P*≤.0001 (highly significant, HS)

**Table 13:** Distribution of bone removal at treated, adjacent and nonadjacent sites

Bone removal in mm	Treated site		Adjacent site		Nonadjacent site	
	Number of sites	%	Number of sites	%	Number of sites	%
0	1	1.78	5	10.86	16	34.78
1	31	55.35	27	58.69	26	56.52
2	17	30.35	14	30.43	4	8.69
3	4	7.14	–	–	–	–

#### The Student t test

The Student *t* test was used to determine whether there was a statistical difference between the groups with regard to the parameters measured.

Student *t* test is as follows:

t=x1−x2s1n1+1n2~~tn1+n2−2, where s2 =n1−1s12 +n2−1s22n1+n2−2

#### One-way analysis of variance (ANOVA)

ANOVA was used to test the difference between groups. Comparison of two variances S_a_^2^ and S_b_^2^ estimated for subjects of the two groups N_a_ and N_b_, respectively, was done using ‘F’ test:

$$F\;=\;\frac{Sa^{2}}{Sb^{2}},\;\mathrm{with}\;N_{a}-1\;\mathrm{and}\;N_{b}-1\;\mathrm{being}\;\mathrm{degrees}\;\mathrm{of}\;\mathrm{freedom}.$$

In the above test, a *P* value less than .05 was accepted as indicating statistical significance. Data analysis was carried out using the Statistical Package for Social Sciences (SPSS, version 10.5).

## RESULTS

Fifteen patients completed this study, and no complication related to the surgery or prosthetic treatment was observed. By the end of the study, all treated teeth were restored with a fixed prosthesis.

### Plaque and gingival indices

No significant change was noted in the plaque index (PI) or gingival index (GI) at the treated, adjacent and nonadjacent sites. The mean values of PI and GI ranged from 1.2 to 1.53 and 1.2 to 1.33, respectively.

### Free gingival margin

The mean distances from the reference stent to the FGM at baseline, 1, 3 and 6 months for TT, AD and NAD sites are listed in Table 1. At all sites, there was a difference in the apical displacement of the free gingival margin from baseline to 1, 3 and 6 months (*P*<.0001), which was highly statistically significant. The location of the FGM at the TT sites was on an average 1.40 and 1.27 mm more apical when compared to that at the AD and NAD sites at 6 months [Tables 1, 2].

### Attachment level

The mean distances from the reference stent to the base of the sulcus at baseline, 1, 3 and 6 months for TT, AD and NAD sites are listed in Table 3. There was an apical shift in the base of the sulcus at all sites from baseline to 1, 3 and 6 months (*P*<.0001), which was highly statistically significant. The attachment loss was greater at TT sites at baseline compared to that at AD and NAD sites at 3 and 6 months [Tables 3, 4].

### Probing depth

The mean probing depths at baseline, 1, 3 and 6 months for TT, AD and NAD sites are listed in Table 5. At treated sites, there was a decrease in the mean probing depth from baseline to 6 months, which was statistically significant (*P*=.001) Probing depth [Tables 5, 6].

### Bone level

The mean distances from the reference stent to the bone level at baseline, 1, 3 and 6 months for TT, AD and NAD sites are listed in Table 7. At all sites, the apical shift in the bone level was different from baseline to 1, 3 and

6 months (*P*<.0001), which was highly statistically significant. The changes in the bone level at TT, AD and NAD sites at 1, 3 and 6 months are listed in Table 8, respectively. At 3 and 6 months, the bone level at treated sites was at a more apical position at baseline when compared to nonadjacent sites [Tables 7–13].

The change in the direct bone level before and after osseous resection at TT, AD and NAD sites was 1.60, 1.06 and 1.00 mm, respectively [Table 11]. The distribution of bone removed during surgical crown-lengthening is listed in Table 13. Overall, the amount of bone resected ranged from 1 to 3 mm.

### Biological width

The mean vertical dimensions of the biological widths at baseline and at 1, 3 and 6 months for TT, AD and NAD sites are listed in Table 9. However, there was no significant difference in the biological width at all sites at 6 months compared to that at baseline (*P*=.817), which was statistically not significant.

At TT, AD and NAD sites, the changes in the biological width at 3 and 6 months are listed in Table 10, respectively.

## DISCUSSION

Preservation of a healthy periodontium is critical for the long-term success of a restored tooth. The term *biological width* is familiar to most clinicians, yet there still exists confusion regarding its meaning and relevance to clinical procedure. The concept of biological width stems from a histological description of the dentogingival complex by Garguilo *et al*.[5]

The primary objective of the crown-lengthening procedure is restoration of an adequate biological width and creation of an adequate space for the proper placement of prosthetic margins. This can be achieved surgically or orthodontically or by a combination of both.

Most authors agree that a minimum distance of 3 mm is required from the osseous crest to the final restorative margin following a crown-lengthening procedure to allow the margin to finish supragingivally.[6] Thus 3 mm allows for 1 mm of supracrestal connective tissue attachment, 1 mm of junctional epithelium and 1 mm for sulcus depth. It should be noted, however, that 3 mm assumes a biological width of approximately 2.04 mm, based on Garguilo’s finding.

Wagenberg *et al*.,^7^ in fact, suggested a 5-mm distance from bone to restorative margin. They clarified that the length of the clinical crown, furcation locations and esthetic considerations limit surgery. Others have also advocated allowing 5 mm from bone to restorative margin to ensure adequate osseous reduction. It is felt that 5 mm will allow for individual variations in biological width dimensions and will prevent the clinician from removing too little bone.

### Changes in the free gingival margin measurement

In this study, there was a significant apical displacement in the free gingival margin at 1, 3 and 6 months. A similar finding has been reported in the respective studies by Bragger *et al*.[6] and Lanning *et al*.[8] There was a greater percentage of apical shift in the free gingival margin position at the treated sites from baseline when compared with adjacent and nonadjacent sites at 6 months, which was statistically significant (*P*≤.0001). This result coincides with the findings of Lanning *et al*.[8]

### Changes in attachment level measurements

There was a statistically significant apical shift in the base of the sulcus at all sites from baseline to 6 months, and this is in agreement with that reported by Lanning *et al*.[8] and Bragger *et al*.^6^

There was no significant difference in attachment level at all sites from 1 to 6 months. The attachment loss was greater at the treated sites from baseline when compared with adjacent and nonadjacent sites at 6 months and is similar to that reported by Lanning *et al*.[8]

### Changes in probing depth

At all sites, there was a decrease in the mean probing depth from baseline to 6 months. This reduction was not statistically significant at the adjacent and nonadjacent sites, which is in agreement with that reported by Lanning *et al*.^8^ At the treated sites, there was a statistically significant reduction in probing depth at 6 months (*P*≤.001), and this is not in agreement with the results observed by Lanning,[8] Pontoriero, Carnevale[9] and Bragger *et al*.^6^

This may be because of greater apical shift in the free gingival margin at treated sites when compared with adjacent sites and nonadjacent sites.

### Changes in bone level

The amount of bone resected in this study was ≥2 mm in 30.35% of treated sites. This could have contributed to the greater percentage of apical shift seen in the free gingival margin at the treated sites when compared with adjacent and nonadjacent sites. This result is similar to that reported by Lanning.[8]

The amount of bone resected at the treated sites was based on the location of the intended prosthetic margin and the original magnitude of the biological width, and this is in agreement with the finding by Smukler *et al*.^10^

The magnitude of bone resected in this study was greater than that in the previous studies by Bragger *et al*.^6^ and Pontoriero and Carnevale.[9] This has contributed to greater stability in the free gingival margin position at treated sites at the end of 6 months. This is in agreement with the finding by Pontoriero *et al*.^9^ and Deas *et al*.^11^

### Changes in the biological width

At all sites, there was a difference in the biological width from baseline to 1 and 3 months; but at the end of 6 months, the biological width was reestablished to its original vertical dimension at all sites without significant difference in its value from baseline. This result is similar to that obtained by Lanning[8] and Oakley *et al*.[12] This could be attributed to the slight gain in the attachment level and apical displacement of the bone level.

At the treated sites, the biological width at 1 and 3 months was significantly different compared to baseline; however, at 6 months, there was no significant difference compared to baseline. In other words, the original vertical dimension of the biological width was reestablished at treated sites 6 months following surgical crown-lengthening. This may be due to the surgical technique, since greater amounts of bone were resected at treated sites compared to adjacent and nonadjacent sites, creating more supracrestal tooth structure.

## SUMMARY AND CONCLUSION

The main objective of this study was to evaluate the positional changes of the periodontal tissues, particularly the biological width, following surgical crown-lengthening in human subjects.

The results showed that there was a significant apical displacement in the free gingival margin at the treated sites, which provided adequate exposure of the crown tooth structure to be restored without impinging on the biological width. There was no statistically significant difference in biological width at all sites. The biological width was reestablished to the original vertical dimension at all sites. Sufficient space was provided coronal to the alveolar crest for the reconstruction of the supracrestal connective tissue.

## References

[1] Padbury A, Eber R, Wang HL (2003). Interactions between the gingiva and the margin of restorations. J Clin Periodontol.

[2] Luis Antonio Fellippe, Monteiro, Luis Clovis, Cardoso Viera, Elito Araujo (2003). Reestablizing biologic width with forced eruption. Quitessence Int.

[3] Gracis S, Fradeani M, Celletti R, Bracchetti G (2000). Biological interaction of aesthetic restorations: factors influencing appearance and long term success. Periodontol.

[4] Bensimon GC (1999). Surgical crown lengthening procedure to enhance esthetics. Int J Periodontics Restorative Dent.

[5] Garguilo AW, Wentz FM, Orban B (1961). Dimensions and relations of the dentogingival junction in humans. J Periodontol.

[6] Bragger U, Lauchenauer D, Lang NP (1992). Surgical lengthening of the clinical crown. J Clin Periodontol.

[7] Wagenberg BD, Eskow RN (1989). Exposing adequate tooth structure for restorative dentistry. Int J Periodontics Restorative Dent.

[8] Lanning SK, Waldrop TC, Gunsolley JC, Maynard JG (2003). Surgical crown lengthening: evaluation of the biological width. J Periodontol.

[9] Pontoriero R, Carnevale G (2001). Surgical crown lengthening:A 12-month clinical wound healing study. J Periodontol.

[10] Smukler H, Chaibi M (1997). Periodontal and dental considerations in clinical crown extension: A rational basis for treatment. Int J Periodontics Restorative Dent.

[11] Deas DE, Moritz AJ, McDonnell HT, Powell CA, Mealey BL (2004). Osseous surgery for crown lengthening: A 6-month clinical study. J Periodontol.

[12] Oakley E, Rhyu C, Karatzas S, Nevins M, Caton J (1999). Formation of the biological width following crown lengthening in non-human primates. Int J Periodontics Restorative Dent.

